# Mining and characterization of ubiquitin E3 ligases expressed in the mouse testis

**DOI:** 10.1186/1471-2164-13-495

**Published:** 2012-09-19

**Authors:** Xiaojun Hou, Wei Zhang, Zhenyu Xiao, Haiyun Gan, Xiwen Lin, Shangying Liao, Chunsheng Han

**Affiliations:** 1 State Key Laboratory of Reproductive Biology, Institute of Zoology, Chinese Academy of Sciences, Beijing, 100101, China; 2 Graduate University of Chinese Academy of Sciences, Beijing, 100049, China

**Keywords:** Spermatogenesis, E3 ubiquitin ligase, Gene mining, Microarray, Testis

## Abstract

**Background:**

Ubiquitin-mediated protein modification and degradation are believed to play important roles in mammalian spermatogenesis. The catalogues of ubiquitin activating enzymes, conjugating enzymes, and ligases (E3s) have been known for mammals such as mice and humans. However, a systematic characterization of E3s expressed during spermatogenesis has not been carried out.

**Results:**

In present study, we set out to mine E3s from the mouse genome and to characterize their expression pattern, subcellular localization, and enzymatic activities based on microarray data and biochemical assays. We identified 398 putative E3s belonging to the RING, U-box, and HECT subfamilies and found that most genes were conserved between mice and humans. We discovered that 73 of them were highly or specifically expressed in the testes based on the microarray expression data. We selected 10 putative E3 genes to examine their mRNA expression pattern, and several genes to study their subcellular localization and E3 ligase activity. RT-PCR results showed that all the selected genes were predominately expressed in the testis. Some putative E3s were localized in the cytoplasm while others were in both the cytoplasm and the nucleus. Moreover, all the selected proteins were enzymatically active as demonstrated by *in vitro* and *in vivo* assays.

**Conclusions:**

We have identified a large number of putative E3s that are expressed during mouse spermatogenesis. Among these, a significant portion is highly or specifically expressed in the testis. Subcellular localization and enzymatic activity assays suggested that these E3s might execute diverse functions in mammalian spermatogenesis. Our results may serve as an initial guide to the field for further functional analysis.

## Background

Spermatogenesis, a complex yet highly regulated developmental process of male germ cells, consists of three stages--the proliferation of spermatogonia, the meiosis of spermatocytes and the morphogenesis of spermatids [1,2]. The formation of the terminally-differentiated germ cells, the spermatozoa, requires a series of important processes that are unique to spermatogenic cells, including nuclear condensation, mitochondrial rearrangement, histone replacement by transition proteins and protamin, the shedding of residual bodies, and the formation of acrosomes [3]. It is not surprising that a large number of testis-specific genes are required for these events. In mice and rats, several high throughput gene expression datasets have revealed that a large number of genes are expressed to fuel spermatogenesis [4,5]. It is also estimated that about 4% of protein-coding genes in the mouse genome are specifically expressed in the mouse testis [6].

Ubiquitination of protein is an indispensable post-translational modification that serves as a component of the protein quality control system. It is also involved in diverse biological processes such as signal transduction, DNA repair, transcriptional regulation in a protein degradation-independent way [7]. In principle, ubiquitination is a process containing three ubiquitin transferring reactions catalyzed by three corresponding enzymes. At first, the 8.5-kDa ubiquitin polypeptide is activated by the ubiquitin-activating enzyme (E1) in an ATP-dependent way. Subsequently, this activated ubiquitin is transferred to the ubiquitin conjugating enzyme (E2) via a thioester bond. In the end, the ubiquitin is transferred to a lysine residue of the substrate catalyzed by the ubiquitin-protein ligase (E3) [8]. Substrate specificity is conferred by E3s, implying a much larger number of E3s in the genome than E1s and E2s. The mammalian genome encodes 1 ~ 2 E1s, 10 ~ 20 E2s and several hundred E3s [9]. There are three typical E3 families including the RING finger family, HECT domain family and the U-box family [10-12].

To our knowledge, approximate 29 E3s have been reported to be expressed in mammalian testes and most of them execute diverse functions at different stages of spermatogenesis (summarized in Additional file 1). A dozen of E3s as well as associated complex play important roles in DNA double strands break (DSB) and histone modifications during early meiosis. As examples, the expression of LASU1 complex and Cul4A-CRL4 is detected primarily in spermatogonia stage and involved in histone ubiquitination during meiosis [13-16]. An N-end rule pathway E3 ligase, UBR2, plays a critical role in transcriptional chromosome inactivation via ubiquitination of histone H2A [17]. The majority of E3s that are highly expressed in haploid germ cells probably contribute to the morphogenesis during the formation of spermatozoa. For instance, MARCH10 is a microtubule associated E3 and is involved in the organization and maintenance of the flagella of spermatozoa [18].

Although it has been known that the mammalian genome encodes a large number of E3s, the exact number is still not known particularly when higher specificity of mining is a major concern to the experimental scientists. Moreover, how these putative E3s are expressed during mammalian spermatogenesis is still an open question. In the present study, we mined out putative E3s from the mouse and human genome, evaluated their expression in multiple tissues, particularly in the mouse testis at different stages. The ligase activity of selected E3s was confirmed by *in vitro* and *in vivo* assays. Our list of E3s expressed during spermatogenesis provides a valuable source for future functional studies of the ubiquitination during mammalian spermatogenesis.

## Results

### Mining of E3s from the mouse and human genomes

**Figure 1:**
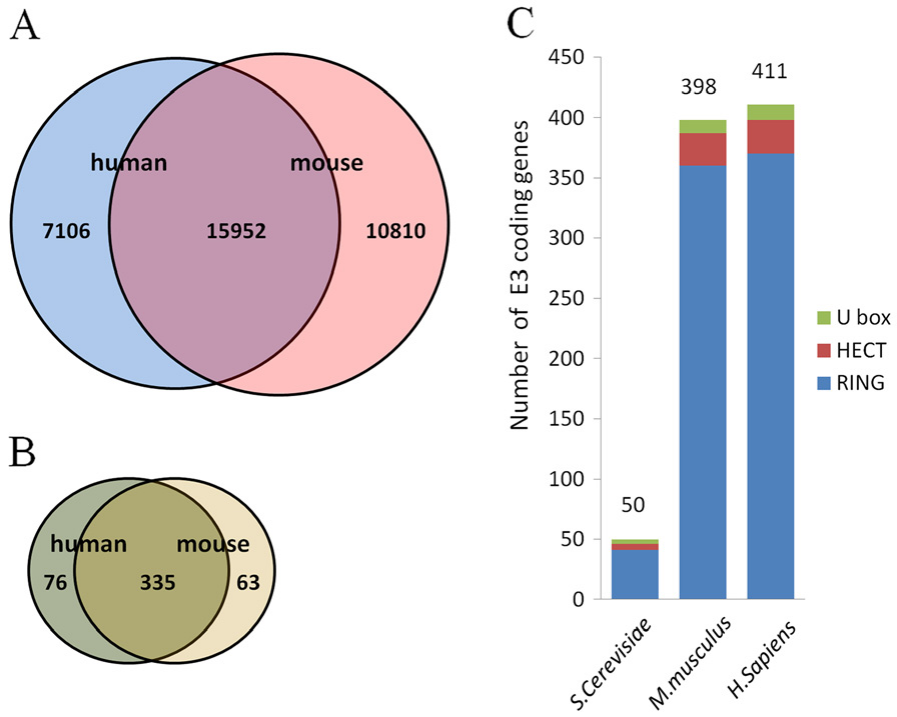
**Numbers of putative E3s mined from the mouse and human genomes and the homologous genes between the two species.** Gene list of protein-coding genes was compiled from lists of genes that can be detected using the Affymetrix microarray technology. Sequences of these genes were downloaded from NCBI Refseq database and were scanned for RING/U-box/HECT domains using the HMMER software. Homologue identification was based on the annotation of NCBI HomoloGene database. (**A**) Number of homologues of all protein-coding genes in humans and mice. The number in the intersection is the number of homologues while the other two represent genes unique to the two species. (**B**) Number of homologues of putative E3 genes in humans and mice. (**C**) Numbers of different E3 genes in humans, mice and yeasts.

To mine putative E3s from the mouse and human genomes, we first compiled all protein-coding genes from several microarray datasets (GSE96, GSE97, GSE1133, GSE2361,GSE9954, GSE1986) generated from gene expression profiling of multiple tissues and the EST dataset from the UniGene database [19-22]. As a result, 26762 and 23058 mouse and human genes were identified, respectively, and 15952 genes were homologous genes (47.1%) based on the HomoloGene database annotation (Figure 1A). We then searched all protein coding genes for domains/motifs in the Pfam_ls and Pfam_fs library (release 23.0) using HMMER 2.3.2 software package (http://hmmer.janelia.org/) with most of the parameters being set to the default values. The E value of the hit was set to be no more than 0.1. Proteins containing the RING domain (zf-C3HC4), the HECT domain and the U-box domain were considered as typical E3s. As a result, 398 and 411 putative E3s were identified from the mouse and the human genome, respectively (see Additional file 2 for mouse E3s). Among them, 335 putative E3s were homologues (70.7%) between the two species (Figure 1B, Additional file 3). The proportion of homologues in the E3 set is significantly higher than expected from the number of general homologues of the mouse and the human genomes (P-value is 3.8E-29 based on the binomial test). We also identified the yeast homologues of the initial 335 mouse/human homologous pairs from 50 yeast E3s (Figure 1C), and only 7 highly conserved E3s among these three species were found (Table 1). These observations suggested that E3 genes are more conserved between the mouse and human genome. 

**Table 1:** E3s conserved in the yeast, the mouse and the human

**Human**			**Mouse**			**Yeast**		
**GeneID**	**Symbol**	**Chr.**	**GeneID**	**symbol**	**Chr.**	**GeneID**	**Symbol**	**Chr.**
10277	UBE4B	1	63958	Ube4b	4	851337	UFD2	IV
4331	MNAT1	14	17420	Mnat1	12	852071	TFB3	IV
9921	RNF10	12	50849	Rnf10	5	851147	MAG2	XII
56852	RAD18	3	58186	Rad18	6	850430	RAD18	III
8315	BRAP	12	72399	Brap	5	856376	ETP1	VIII
5192	PEX10	1	668173	Pex10	4	851858	PEX10	IV
23327	NEDD4L	18	83814	Nedd4l	18	856862	RSP5	V

### Expression of E3 mRNAs in the mouse testis

**Figure 2:**
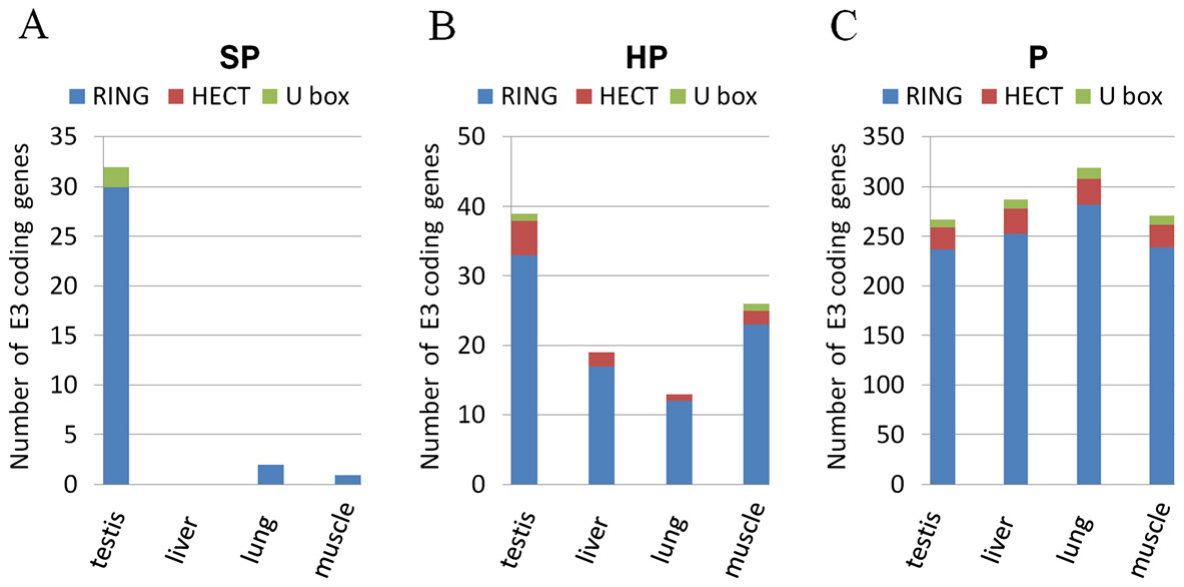
**Expression profiles of E3 genes in different tissues.** Expression of different families of E3s were ranked based on their levels in the mouse testis (P for present, HP for highly present, SP for specific). The expression of genes of these three levels were examined in other tissues such as the liver, the lung, and muscle. (**A**) Almost all the SP-level E3 genes were only expressed in the mouse testes. (**B**) More HP-level genes were expressed in the testes than in other tissues. (**C**) Genes that were expressed in the testis (P-level genes) were also expressed in other tissues.

We next examined the mRNA expression levels of E3 genes in the mouse based on four microarray datasets (GSE97, GSE1133, GSE9954, and GSE1986) and the UniGene dataset. For each dataset, we defined 5 expression levels of an mRNA in a particular tissue in the increasing order of tissue specificity—A (absent), P (present), HP (highly present), MS (multiple tissue-specific), and SP (specific). The level A/P is just based on a gene’s P/A call value determined by the Affymatrix platform using the MAS5 algorithm [23]. The other three levels were based on the comparison of the mRNA’s z-score with three threshold values (See Methods for further explanation). The evaluations from different datasets were summarized by voting—A level value is assigned only when it is supported by no less than two datasets. A putative E3 is always assigned to the highest specificity group exclusively. As a result, we identified 267 P level E3s, 39 HP level E3s, 2 MS level E3s, and 32 SP level E3s in the mouse testis (Table 2). Compared with other tissues such as the liver, the lung, the muscle, the testis contains significantly more specific E3s (Figure 2). Therefore, a large number of E3s are expressed in the mouse testis and a significant portion is specifically expressed. 

**Table 2:** Numbers and mRNA expression levels of annotated E3s in the mouse testis

	**SP**	**MS**	**HP**	**P**	**A**	**Total**
zf-C3HC4	30	2	33	237	58	360
HECT	0	0	5	22	0	27
U box	2	0	1	8	0	11
total	32	2	39	267	58	398

### Stage-specific expression of E3 mRNAs during mouse spermatogenesis

We further studied the stage-specific expression of putative E3s during mouse spermatogenesis using three microarray datasets (GSE640, GSE926 and GSE4193) derived from gene expression profiling of testis at different days postpartum or of different spermatogenic cell types [6,24,25]. Genes that are 3-folds or more differentially expressed at one or more time point (or cell type) were selected for further clustering analysis. Based on visual inspection of the heat maps of the clustering analysis, three clusters of genes were identified according to the stages when their expressions start to increase. Genes in group MI are expressed at a higher level at the mitotic division stage, genes in group ME start to increase their expression level at meiotic division stage while genes in group PM start to increase their expression at the post meiosis stage. Results from the three datasets were again summarized by voting—a gene was assigned to a particular group only if it was supported by 2 or 3 votes. As results, 20, 24 and 6 E3s were assigned to the MI, ME, and PM groups, respectively, totaling up to 50 (Table 3 and Additional file 4). By taking intersections of each of three groups with each of the SP, MS, HP, and P classes, it can be seen that the putative testis specific E3s increase their expression during or after meiosis. 

**Table 3:** Numbers of stage-specifically expressed E3 mRNAs during mouse spermatogenesis

**Cell type**	**Testis expression level**				**Total**
	**SP**	**MS**	**HP**	**P**	
MI	0	0	2	18	20
ME	13	0	7	4	24
PM	5	0	0	1	6
	18	0	9	23	50

### Stage-specific expression of E3 proteins during mouse spermatogenesis

**Figure 3:**
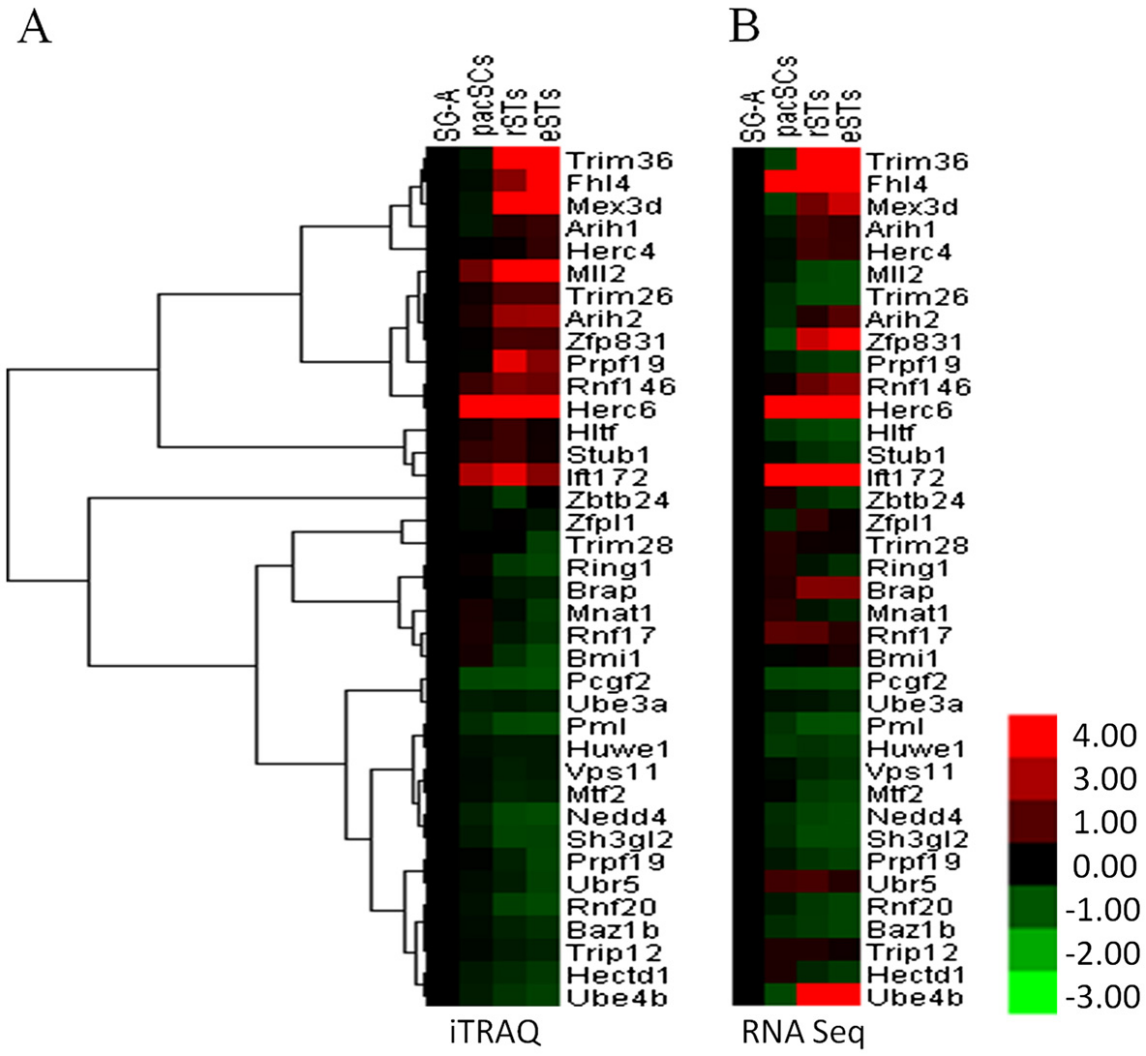
**Putative E3s whose proteins were detected in one or more germ cell types using the iTRAQ mass spectrometry and their corresponding RNA levels detected by RNA-seq data.** The protein and RNA expression data of these putative E3s were from two independent studies aiming to examine global gene expression at the mRNA and protein levels. Note most putative E3s were expressed at or after meiosis. The mRNA expression either matches or precedes their protein expression. SG-A: type A spermatogonia, pacSC: pachytene spermatocyte, rST: round spermatid, eST: elonged spermatid.

In a separate study, we examined protein levels in type A spermatogonia, pachytene spermatocytes, round spermatids, and elongated spermatids using the iTRAQ mass spectrometry (unpublished data). Thousands of proteins were identified in each cell type. We selected E3s from these detected proteins and examined their dynamic changes during spermatogenesis. 38 annotated E3s were found (Additional file 5). The heat maps of the clustering analysis showed that these proteins could be separated into two groups according to the dynamic change of their expression. Protein expression of group 1 is up regulated after meiosis while group 2 is on the contrary. Meanwhile, we performed an RNA deep sequencing in the same cell type (unpublished data). Compared to iTRAQ analysis, the expression patterns of most RNAs were consistent with those of protein expression. The changes of a portion of proteins were lagging behind those of their RNAs probably due to translation inhibition (Figure 3).

### Validation of mRNA expression of selected putative E3s by RT-PCR

**Figure 4:**
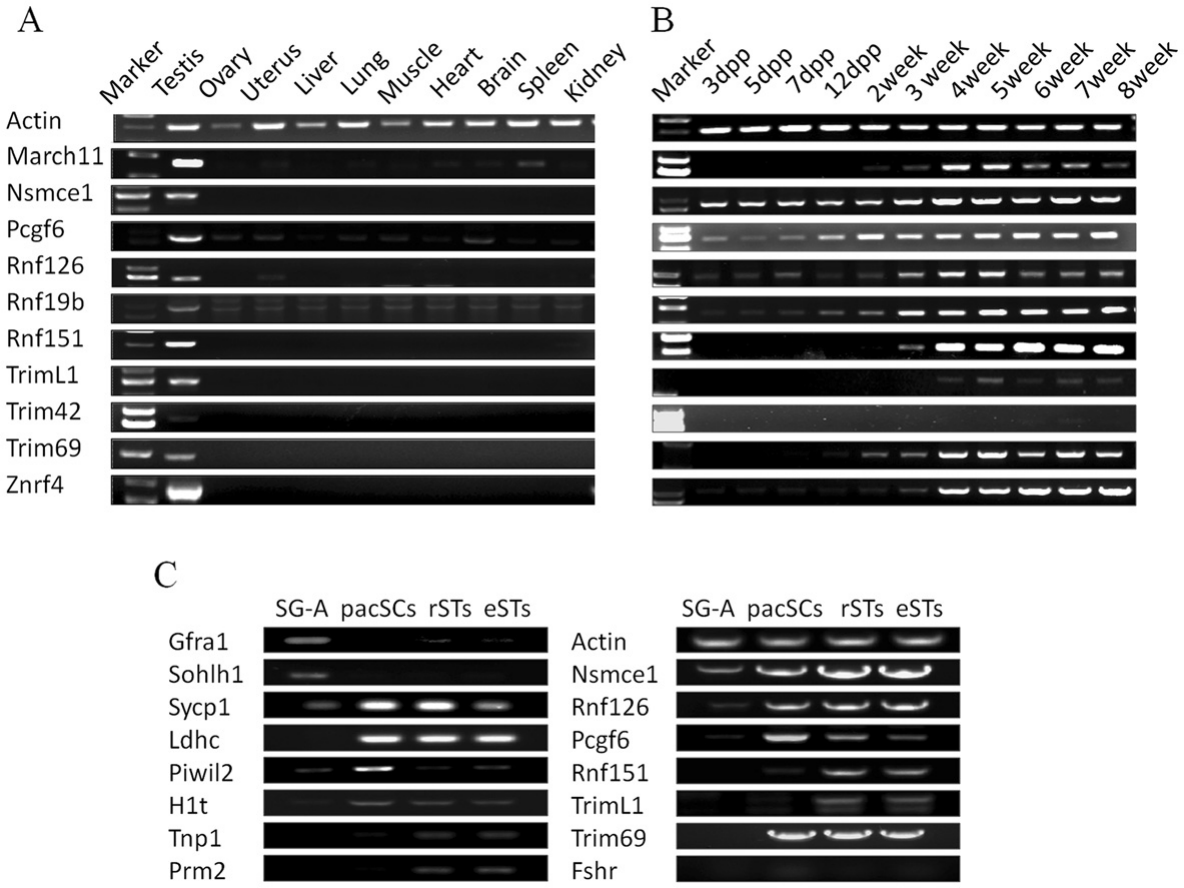
**Examination of mRNA expression of annotated murine testis-specific E3s by RT-PCR.** (**A**). mRNA expression in 10 tissues. (**B**). mRNA expression in testis of different days post partum. (**C**). mRNA expression in 4 isolated germ cell types.

Among the 32 putative testis-specific E3s, 19 have not been well studied. We selected 10 of them randomly and checked their mRNA expression in 10 different tissues (testis, ovary, uterus, liver, lung, muscle, heart, brain, spleen and kidney) using RT-PCR. As shown by Figure 4A, majority were exclusively detected in the testis while the remaining one were expressed in the testis at much higher levels than in other tissues. These result indicated that our testis specificity evaluation of genes based on microarray data is highly reliable. Examination of their expression at different days postpartum indicated that almost all of them were expressed at stages when haploid cells are generated (Figure 4B). We further examined expression of some genes in isolated type A spermatogonia (SG-A), pachytene spermatocyte (pacSCs), round spermatid (rSTs) and elonged spermatid (eSTs). The purity of the isolated germ cells all exceeded 90% as determined by morphological evaluation, and was also confirmed by measuring the expression of known marker genes that are either uniquely or highly expressed in each type cells (*Gfrα*1 and *Sohlh1* for type A spermatogonia; *Ldhc* for germ cell except spermatogonia; *Sycp1*, *Piwil2* and *H1t* for spermatocytes; *Tnp1* and *Prm2* for spermatids; *Fshr* for sertoli cell). As shown by Figure 4C, most of the E3s start their expression in either pacSCs or rSTs.

### Subcellular localization of putative E3s with or without transmembrane domains

**Figure 5:**
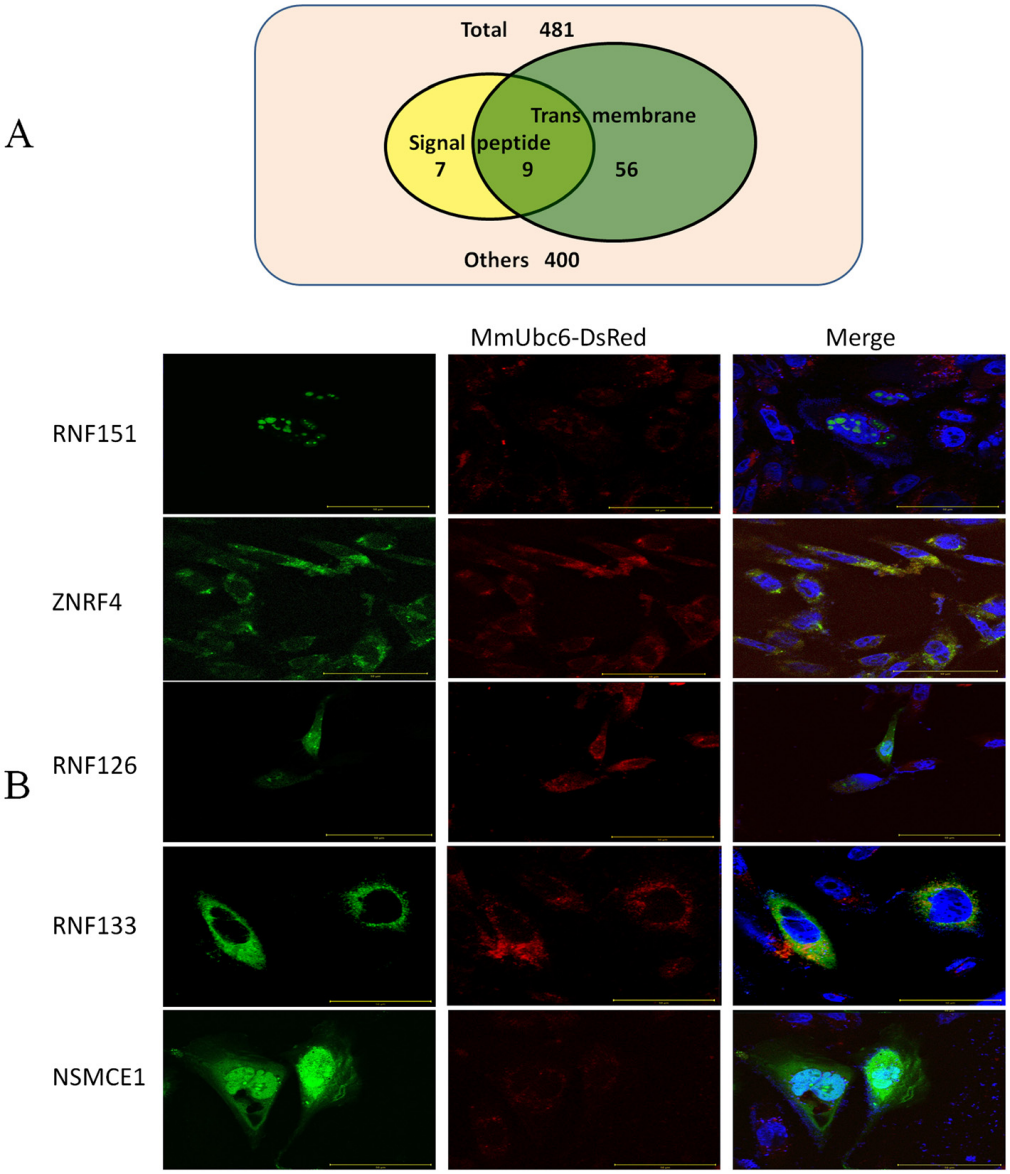
**Analysis of E3s containing signal peptide and transmembrane domains (TMDs).** (**A**) Number of E3s which possessed signal peptides and TMDs. (**B**) CHO cells were transiently transfected with GFP tag recombinant plasmids. The cells were then processed for immunofluorescence microscopy 24 hours after transfection and MG-132 treatment. The scale bar is 50 μm.

A significant number of E3s have signal peptides and/or transmembrane domains (TMD) suggesting that they may anchor to the cell’s membrane system to execute their functions. 16 and 65 annotated E3s were identified with either signal peptide and/or TMD, respectively (Figure 5A). As subcellular localizations of E3s help to understand their function, and no antibodies were available for *in vivo* studies, we decided to transfect CHO cells with plasmid constructs of E3s fused to EGFP to study their subcellular localization. We selected 2 E3s with TMD (RNF133, ZNRF4) and 3 without (RNF151, RNF126, NSMCE1), and used mouse Ubc6, an endoplasmic reticulum-localized integral membrane E2, as the reference [26]. CHO cells were co-transfected with the EGFP-E3 and the MmUbc6-DsRed constructs. Subcellular localization of the fusion proteins were inspected under fluorescent microscope. As shown in Figure 5B, RNF151 was only localized in the nucleus in a punctate manner. RNF126 and NSMCE1 was localized both in the nucleus and the cytoplasm in a diffused manner, while RNF133 and ZNRF4 were only localized in the cytoplasm, co-localized well with MmUbc6-DsRed. The localizations of RNF133 and ZNRF4 were consistent with previous report [27,28], indicating that our assays using the fluorescent fusion proteins were reliable for subcellular localization studies. 

### *In vitro* and *in vivo* ubiquitin ligase activities of putative E3s

**Figure 6:**
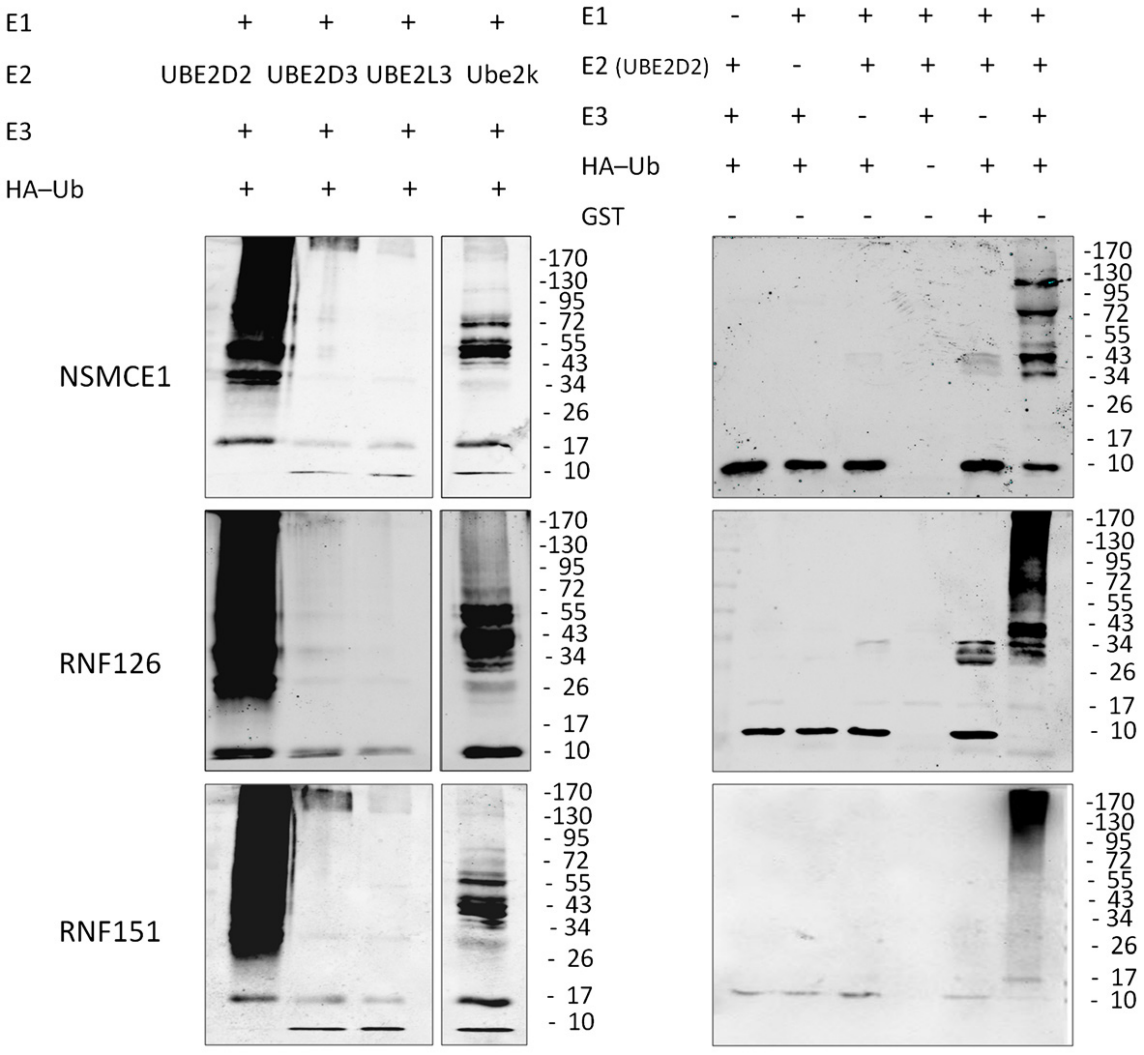
**Ligase activity of putative E3s was validated by* in vitro *assays.** Polyubiquitin chains were formed in the presence of UBE2D2 and Ube2k. Polyubiquitin chains were observed only in the presence of E1, E2, E3 and ubiquitin. GST protein was used as negative control (right lane6).

It was important to test whether the putative E3s indeed possess ubiquitin ligase activities in order to understand their function. However, due to the lack of antibodies to immunoprecipitate native E3s from testis or germ cell lysate, we decided to used recombinant protein and tagged protein immunoprecipitated from transfected cells to examine their enzymatic activity. Three putative E3 genes (*Nsmce1*, *Rnf126* and *Rnf151*) were cloned into the pGEX-4T1 vector. GST fusion proteins were expressed and purified from *E coli*. In vitro ubiquitination assays were performed by including E1, E2, E3, and ubiquitin. Anti-Ubiquitin (Ub) antibody was used to detect polyubiquitin chains. To screen the E2 with highest ubiquitin catalytic activity, three human and one mouse recombinant E2s (UBE2D2/human, UBE2D3/human, UBE2L3/human and Ube2k/mouse) were tested. Our results showed that UBE2L3 has barely-detectable activity while UBE2D2 has the highest activity (Figure 6). The polyubiquitin chain was formed when all reactants were added (E1, E2, E3, and ubiquitin), while, it was not detected if any of the reactants were excluded. Therefore, the ligase activity of the putative E3s was detected in a substrate-independent way.

**Figure 7:**
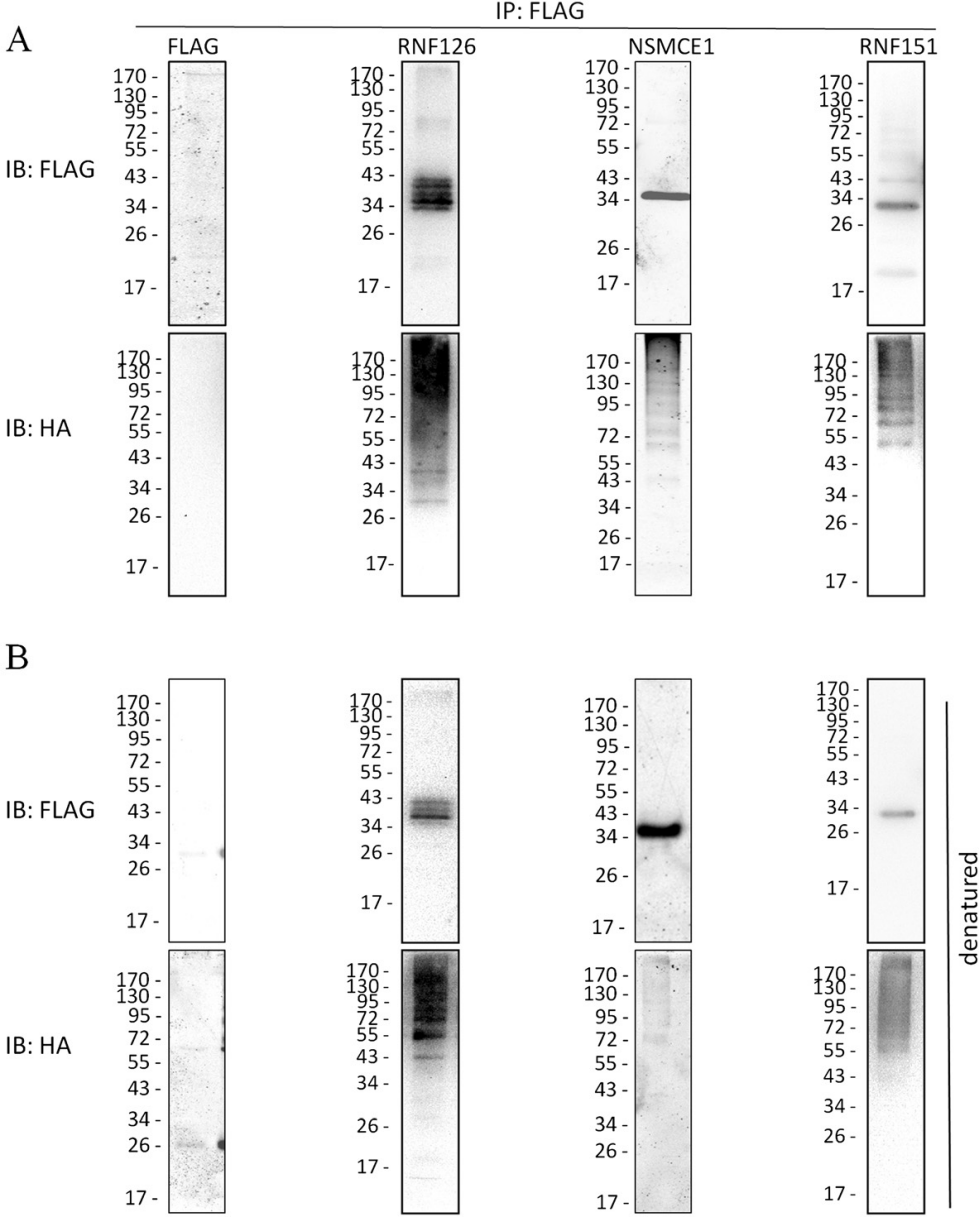
**Ligase activity of putative E3s was validated by* in vivo *assays.** (**A**) HEK 293FT cells were transfected together with FLAG-E3s and HA-Ub. After MG-132 treatment, whole cell lysate was subjected to immunoprecipitation with anti-FLAG beads. Poly-ubiquitination was detected by Western blotting with anti-FLAG and anti-HA antibody. (**B**) Protein mixture was subjected to anti-FLAG beads after heating with 1% SDS.

The ligase activity of these E3s was also confirmed in HEK 293FT cells transfected with plasmid constructs that gave rise to FLAG-tagged E3s and HA-tagged Ub. Cell lysate was subjected to immunoprecipitation (IP) using the FLAG antibody conjugated beads and immunoblotted using the anti-HA antibody. As shown in Figure 7A, poly (Ub) chains were detected for all tested E3s. It has been known that some E3s are first polyubiquitinated before they transfer the poly (Ub) chain to their substrates. To tell whether the poly (Ub) chain detected was from the E3s or any protein that was co-immunoprecipitated with the E3s, the cell lysate was first boiled with high concentration SDS to disrupt any potential protein-protein interaction before immunoprecipitated with the beads. As shown in Figure 7B, polyubiquitination was still detected with RNF126 and RNF151. These results showed that some E3s promoted self-ubiquitination before their substrates were ubiquitinated. A potential role of self-ubiquitination of E3s is to regulate the ligase activity and recruit substrates during spermatogenesis [29]. Our results also indicated that it was very likely that most, if not all, E3s identified by bioinformatics analysis were active enzymes. Considering their different sub-cellular localizations as indicated by the transfection assays, it seemed that these E3s were involved in diverse molecular processes during spermatogenesis such as protein quality control, organelle turnover, chromatin remodeling, to name a few. 

## Discussion

In the present study, we conducted genome-wide search and annotation of putative E3s from several high-throughput expression datasets. We mined out putative E3s from protein coding genes whose expression have been examined in different tissues, and investigated their mRNA and protein expressions in the mouse testis. According to microarray data, we discovered that a large number of putative E3s were expressed in the mouse testis and that some of them were specifically expressed there, which were confirmed by RT-PCRs. The protein expression patterns of 38 E3s were detected in different spermatogenic cells using the iTRAQ MS technology (our unpublished data). The E3 ligase activity of some selected proteins was confirmed by ubiquitin assays. These results indicated that our mining and annotation strategy was reliable and distinguished its self from some previous mining procedures purely based on sequence analysis.

According to a report by Li et al. based on bioinformatics analysis, the human genome contains 617 putative E3s [9]. This number was derived from motif searching using the Hidden Markov Models (HMMs) for domains such as HECT, RING/U-box, F-box, SOCS-box etc. It is known that most, if not all, HECT and RING/U-box proteins are true E3s while those F-box-, SOCS-box containing ones are not necessarily a component of the E3 complexes [30]. Therefore, we limited our searches by using only the HECT and RING/U-box domains to reduce the false positive rate. Indeed, Li et al. used several datasets containing predicted transcripts and corresponding proteins of the human genome in order for the search to be comprehensive. However, we think for bench-working scientists, higher specificity is more important than higher sensitivity. Accordingly, we only used protein-coding genes derived from the Affymetrix expression GeneChips (Mouse Genome 430 2.0 Array, Murine Genome U74 Version 2 Array, Human Genome U95A Array, Human Genome U133A Array and GNF1M/GNF1H custom array) and the UniGene database as the target sets to be mined. As a result, we identified 398 putative E3s, of which 360 were the RING family members from the mouse genome. Our 411 human E3s are not all covered by Li’s 617 E3s. The intersection between our 411 human E3s and the 617 by Li et al. is 308. We found that the list of Li et al. only contained 343 human E3s based on the HECT and RING/U-box domains. Therefore, our mining should be more specific than Li et al. and also more sensitive if the same set of domains were used. Moreover, we found that Li et al. mined 34 more E3s based on only RING and U-box domains than we did. However, a manual curation of these 34 genes by us indicated that 26 actually did not contain the RING or U-box, 3 were pseudogenes, 1 was discontinued gene, 3 were E4s, and only 1 typical E3 was left.

The main goal of the present study was to profile the expression of E3s in the mouse testis during spermatogenesis. Taking advantage of several multiple tissue microarray datasets, we rated the expression levels of putative E3s as 5 levels (A, P, HP, MS and SP). The rating system was based on a supposed normal distribution of gene expression in different tissues and the final rate was determined by votes from different datasets. Therefore, the rating was highly stringent as was confirmed by the RT-PCR results. Almost all predicted testis-specific E3s were indeed specifically expressed in the testis. In addition, we found that majority of the putative E3s were expressed in the mouse testis, and the number of testis-specific E3s was much higher than in other tissues such as the liver, the lung, and the muscle. The stages-specific expression of E3s during mouse spermatogenesis indicated that most of the testis-specific E3s were expressed in spermatocytes and spermatids. As spermatocytes undergo meiosis and spermatids go through a number of unique processes such as acrosome formation, histone replacement, flagellum formation and shedding of most of the cytoplasm, it is reasonable that these cells need many tissue-specific E3s to catalyze the turnover of proteins.

## Conclusions

We performed a mining of ubiquitin ligases in the mouse genome and analyzed their expression profile in the mouse testis systematically. The identification of a large number of homologous putative E3s between human and mouse suggests that these enzymes are highly conserved in the two species. Our analysis reveals that 340 putative E3s are expressed in the mouse testis and 73 E3s are highly or specifically expressed. Based on *in vitro* and *in vivo* essays, the ligase activity of several novel E3s expressed during the mouse spermatogenesis are confirmed. The lists of E3s expressed at different levels during spermatogenesis provide a valuable resource from which key factors regulating spermatogenesis could be identified.

## Methods

### mRNA expression evaluation in tissues

In order to summarize the expression levels of a gene in a particular tissue (t) from different datasets (d), the expression value of a gene in a tissue (x_t_) were converted into the z-score (z_t_ = (x_t_ - μ)/s where μ and s are the mean and standard deviation of x_t_ among tissues). Assuming the z_t_ values of a gene in different tissues are normally distributed, three threshold values (z_1_ = 1, z_2_ = 1.96, z_3_ = 2.58) mean that at least 68%, 95%, 99% of tissues have the absolute values of their z_t_ scores to be less than these value, respectively. For example, if a gene in the testis has its z_t=testis_ ≥ 2.58, we can say that the z_t≠testis_ values of this gene in at least 99.5% of the tissues should be less than z_t=testis_ while z_t≠testis_ values in at most 0.5% of the other tissues may be equal to or more than z_t=testis_. The following five expression levels were defined accordingly. A for absent of a gene in t if the original Affymetrix P/A call value is A. P for present if the Affymetrix P/A call value is P. HP for highly present with z_t_ ≥ z_1_. MS for multi-tissue specific with z_t_ ≥ z_2_ because a gene with such an expression value may only expressed in several tissues. SP for specific with z_t_ ≥ z_3_ for tissue t and z_i_ ≤ z_2_ for all the other tissues (i ≠ t). The most specific expression level was assigned to a gene if it satisfying more than one level. For example, a gene considered SP must also satisfy the MS condition, but we labeled it as SP. We considered five datasets (including the four mouse microarray datasets and the UniGene EST dataset) as five voters. We determined the mRNA expression level by a ballot. The expression level that was supported by the highest number of votes was assigned to the gene in the specified tissue. However, if two expression levels were both supported by the highest number of votes, the less specific level was assigned. For example, MS was assigned to a gene if both MS and SP were supported by 2 votes.

### Animal use

Male CD1 mice were purchased from Vital River Laboratories (Beijing, China) and maintained in the Experiment Animal Center, Chinese Academy of Sciences. Animal use was approved by the Animal Research Committee of the Institute of Zoology, Chinese Academy of Sciences (Protocol No. 2004–35). Protocols for animal sacrifice and tissue harvesting were in accordance with the NIH Guide for the Care and Use of Laboratory Animals. All the mice were euthanized by cervical dislocation before tissue harvesting.

### Isolation of different type germ cells

8 dpp and 17 dpp mice were used for isolating type A spermatogonia (SG-A) and pachytene spermatocytes (pacSCs), respectively. Meanwhile, adult mice were used for isolating round spermatids (rSTs) and elonged spermatids (eSTs). The seminiferous tubules were isolated from decapsulated testes and digested into cell suspension with collagenase (1 mg/ml, 2 min, 37°C, Gibco) and trypsin (0.25%, 5 min, 37°C, Gibco). The purity of four types of germ cells all exceeded 90% using the unit gravity sedimentation procedure in 2-4% BSA medium as described before [31-33]. The purity of cells was validated based on morphological evaluation and confirmed by RT-PCR of reported marker genes expressed in different germ cells.

### RNA isolation and RT-PCR

Total RNAs were extracted from mouse testes at different developmental stages and other tissues using TRIzol solution. 2 μg RNA was reverse-transcribed into cDNA by reverse transcriptase (Promega). The primers used for PCR are listed in Table 4. PCR reactions were conducted following stand protocol. Briefly, the reactions were started at 94°C for 3 min, and went through 27 cycles with denaturing at 94°C for 30 sec, annealing 60°C (or other suitable Tms) for 30 sec, and elongation at 72°C for 40 sec. Reactions were finished with a final extension step at 72°C for 10 min.

**Table 4:** Primers used in RT-PCR analysis

**Name**	**Product length**	**Sequence (5'-3')**
Action	480 bp	Sense: ATATCGCTGCGCTGGTCGTC
		Antisense: ATATCGCTGCGCTGGTCGTC
March11	321 bp	Sense: ATGAGCGACGAGGGCAAAAAAC
		Antisense: CTACTGAACTTTCTCAACCAGT
Nsmce1	830 bp	Sense: ATATGGCTTCCGGCGTGATC
		Antisense: TTGTGCTGCCGGGTCCGTAA
Pcgf6	698 bp	Sense: AAGCCTGCTGCTCCACA
		Antisense: TGACCCACCCACCTCAA
Rnf126	924 bp	Sense: ATATGGCCGAGGCGTCGCCG
		Antisense: TTGGAGTTGCTTGTGGCG
Rnf19b	407 bp	Sense: AGCGGAAGCCCTACAGA
		Antisense: CCACCACCAGCACCATT
Rnf151	519 bp	Sense: GCCGCCTACAAGTCAAG
		Antisense: GCCTGGGTTCAGTGTTTA
TrimL1	1850 bp	Sense: TGATGTCCAACCATGAGA
		Antisense: CAGATGTGGCAGATGATAAG
Trim42	816 bp	Sense: ACCAATCTGAAGTGCTACTA
		Antisense: CTACAGGTCATTATCAAT
Trim69	1517 bp	Sense: CAAGTTTCATGGAGGTATC
		Antisense: ATCTGTGGATGTACGATGTG
Znrf4	876 bp	Sense: CATGGCGCGGTTTGCGTG
		Antisense: TCAGGAGAGCTCGGAAGTCGCTTC

### Molecular cloning of putative E3s

cDNAs of putative mouse E3s were amplified using PCRs. Restriction enzyme sites were included in the primers. PCR products and vector plasmids were digested with selected restriction enzymes, target fragments were gel purified and ligated subsequently. Full- or partial length cDNAs of putative E3s were sub-cloned into pFLAG-CMV4, pEGFP-N1 and pGEX-4T1 to make different fusion proteins. The primers used for plasmid construction were listed in Table 5. All the recombinant plasmids were confirmed by DNA sequencing. Cloning procedures followed standard protocols.

**Table 5:** Primers used for recombinant plasmids construction

**Name**	**Vector**	**Sequence (5'-3')**
Nsmce1	pGEX-4 T1	Sense: CG GGATCC ATGGCTTCCGGCGTGATC
		Antisense: CG GAATTC CTAGTGCTGCCGGGTCCGTAA
	pEGFP-N1	Sense: CG GAATTC ATATGGCTTCCGGCGTGATC
		Antisense: CG GGATCC TTGTGCTGCCGGGTCCGTAA
	pFLAG-CMV4	Sense: CG GAATTC TATGGCTTCCGGCGTGATC
		Antisense: CG GGATCC CTAGTGCTGCCGGGTCCGTAA
Rnf126	pGEX-4 T1	Sense: CG GGATCC ATGGCCGAGGCGTCGCCG
		Antisense: CG GAATTC TCAGGAGTTGCTTGTGGCG
	pEGFP-N1	Sense: CG GAATTC ATATGGCCGAGGCGTCGCCG
		Antisense: CG GGATCC TTGGAGTTGCTTGTGGCG
	pFLAG-CMV4	Sense: CG GAATTC TATGGCCGAGGCGTCGCCG
		Antisense: CG GGATCC TCAGGAGTTGCTTGTGGCG
Rnf151	pGEX-4 T1	Sense: CCG GGATCC TTCCTGTGTTCTGTCTGCCA
		Antisense: GC CTCGAG CCTTCATAAGACACTTTGGC
	pFLAG-CMV4	Sense: CG GAATTC GATC ATGAGTGGTGG
		Antisense: CG GGATCC TCATAAGACACTTTGGC
Znrf4	pEGFP-N1	Sense: CCG GAATTC CCATGGCGCGGTTTGCGTG
		Antisense: CCG GGATCC CTGGAG AGCT CGGA AGT CGC TTC
	pFLAG-CMV4	Sense: CCG GAATTC CATGGCGCGGTTTGCGTG
		Antisense:CCG GGATCC TCAGGAGAGCTCGGAAGTCGC TTC

### Preparation of recombinant proteins

Putative E3s with GST tag and E2s (UBE2D2, UBE2D3, UBE2L3, Ube2k) with His tag were produced in the *Escherichia coli* strain Rossetta (DE3) (TransGen). Bacterial culture, IPTG treatment and lysate sonication were conducted following standard protocols. GST and His tag fusion proteins were purified by AKTA^TM^ purifier 9500 using GST-Trap_5ml_FF and His-Trap_5ml_FF columns (GE Health care), respectively. Protein purification followed the manufacturer’s instructions. Purified proteins were dialyzed by ultrafiltration (MICROCON, Millipore) before *in vitro* ubiquitination assays were performed.

### *In vitro* ubiquitination assays

*In vitro* ubiquitination reactions were set up by adding to the Eppendorf tubes with the following reactants: 0.25 μg yeast E1 (Boston Biochem); 2 μg various purified recombinant E2s (UBE2D2/UBCH5B, UBE2D3/UBCH5C, UBE2L3/ UBCH7, Ube2k/E2-25K); 5 μg putative E3s with GST tag; 5 μg MYC-ubiquitin (Boston Biochem) in 50 mM Tris/HCl (pH 7.4), 5 mM MgCl_2_, 1 mM DTT; 2 mM ATP (Sigma). Negative control reaction was set up with putative E3 being replaced with equal amount of glutathione S-transferase. After 30 min incubation at 37°C, reactions were stopped by treatment at 95°C for 10 min in a SDS–PAGE sample buffer containing β-mercaptoethanol. Then proteins were separated by electrophoresis and subjected to Western blot using anti-ubiquitin antibody [34].

### Cell culture and transfection

HEK 293FT and CHO cells were cultured in high glucose DMEM (Gibco) supplemented with 10% fetal bovine serum (Hyclone) in 5% CO_2_ at 37°C. 24 hours after passage, 3.5 × 10^4^/cm^2^ cells were plated and transient transfected with Mega Trans1.0 (ORIGEN) according to the manufacturer’s recommendations. 24 hours after transfection, CHO cells were treated with 10 μM MG-132 (Z–Leu–Leu –Leu-al, Sigma) for 6~8 hours before fixed in 4% paraformaldehyde for 15 min at room temperature. Then the cells were permeabilized with 0.1% Triton X-100 and washed by PBS. Cell nucleus was dyed with DAPI (1 μg/ml) for 10 minutes. Samples were observed with Zeiss LSM710 confocal microscope. All the images were quantified using the ZEN software (Zeiss).

### *In vivo* Ubiquitination assays

HEK 293FT cells transfected with different FLAG-tagged putative E3s and HA-tagged PRK-Ub were cultured for 36 hours. Cells were treated with 10 μM proteasome inhibitor MG-132 for 6~8 hours. Subsequently, the cells were washed twice in ice-cold PBS and suspended in cell lysis buffer [50 mM Tris HCl (pH7.4), 150 mM NaCl, 1 mM EDTA, 1% NP-40, and 10 μM protease Inhibitor Cocktail (Sigma)]. The cell lysate (3 × 10^7^ cells/ml, approximately 0.5 ~ 1 mg/ml) was incubated on a shaker for 30 minutes at 4°C and centrifuged at 15000 g for 15 min. 1 ml supernatant was incubated with 30 μl suspended ANTI-FLAG M2 affinity gel (Sigma) overnight at 4°C and then spun at 15,000 g for 15 min [35]. The M2 resin was washed 4 times with buffer A [50 mM Tris–HCl (pH7.4), 150 mM NaCl], bound proteins were eluted using buffer B (including 150 ng/μl 1 × FLAG peptide, Sigma). Eluted proteins were separated by electrophoresis using 4~20% gradient PAGE gels [36,37].

In order to disrupt the non-covalent interactions, cells were lysed with lysis buffer containing 1% (w/v) sodium dodecyl sulfate (SDS) and then heated to 100°C. 10 mM sulfhydryl alkylating agent N-ethylmaleimide (NEM, Pierce) was added into the cell lysis buffer to prevent ubiquitin chain from breaking. The concentrations of SDS in the cell lysis were diluted to 0.1% before immunoprecipitated with the ANTI-FLAG M2 beads [38].

### Western blot analysis

Western blot analysis followed standard protocol. Primary antibodies and dilution factors were as the following: anti-FLAG and anti-HA, 1:5000 (mouse, monoclonal antibody, Abmart); anti-ubiquitin, 1:1000 (mouse, polyclonal antibody, Santa Cruz). The secondary antibody was anti-mouse IgG (goat polyclonal antibody labeled with Dylight 800, 1:5000, KPL). Fluorescent signal on NC-membrane was detected with an Odyssey infrared imaging system (LI-COR Biosciences).

## Competing interests

The authors declare that they have no competing interests.

## Authors’ contributions

CH, XH, and WZ conceived and designed the experiments. XH, WZ, SL and ZX conducted the experiments. WZ, HG and XL analyzed microarray data. XH, and CH wrote the manuscript. All authors have read and approved the final manuscript.

## Supplementary Material

Additional file 1 Summary of published papers about E3s in spermatogenesis. Click here for file

Additional file 2 All the E3 coding genes annotated from the mouse genome. Click here for file

Additional file 3 E3 homologues in the mouse and human. Click here for file

Additional file 4 Stage-specific expressed E3 genes from microarray data. Click here for file

Additional file 5 Expression of annotated E3s from iTRAQ database. Click here for file

## References

[B1] Russell LD Ettlin RA Sinha HAP Clegg ED Russell LD, Ettlin RA, Sinha HAP, Clegg ED Mammalian spermatogenesis Histological and Histopathological Evaluation of the Testis 1990 Cache River Press, St. Louis 1 40

[B2] Dym M Spermatogonial stem cells of the testis Proc Natl Acad Sci U S A 1994 91 24 11287 11289 10.1073/pnas.91.24.11287 7972051 PMC45216

[B3] Hecht NB Molecular mechanisms of male germ cell differentiation Bioessays 1998 20 7 555 561 10.1002/(SICI)1521-1878(199807)20:7<555::AID-BIES6>3.0.CO;2-J 9723004

[B4] Johnston DS Wright WW Dicandeloro P Wilson E Kopf GS Jelinsky SA Stage-specific gene expression is a fundamental characteristic of rat spermatogenic cells and Sertoli cells Proc Natl Acad Sci U S A 2008 105 24 8315 8320 10.1073/pnas.0709854105 18544648 PMC2448834

[B5] Pang AL Johnson W Ravindranath N Dym M Rennert OM Chan WY Expression profiling of purified male germ cells: stage-specific expression patterns related to meiosis and postmeiotic development Physiol Genomics 2006 24 2 75 85 16291737 10.1152/physiolgenomics.00215.2004

[B6] Schultz N Hamra FK Garbers DL A multitude of genes expressed solely in meiotic or postmeiotic spermatogenic cells offers a myriad of contraceptive targets Proc Natl Acad Sci U S A 2003 100 21 12201 12206 10.1073/pnas.1635054100 14526100 PMC218736

[B7] Welchman RL Gordon C Mayer RJ Ubiquitin and ubiquitin-like proteins as multifunctional signals Nat Rev Mol Cell Biol 2005 6 8 599 609 10.1038/nrm1700 16064136

[B8] Hershko A Heller H Elias S Ciechanover A Components of ubiquitin-protein ligase system. Resolution, affinity purification, and role in protein breakdown J Biol Chem 1983 258 13 8206 8214 6305978

[B9] Li W Bengtson MH Ulbrich A Matsuda A Reddy VA Orth A Chanda SK Batalov S Joazeiro CA Genome-wide and functional annotation of human E3 ubiquitin ligases identifies MULAN, a mitochondrial E3 that regulates the organelle's dynamics and signaling PLoS One 2008 3 1 e1487 10.1371/journal.pone.0001487 18213395 PMC2198940

[B10] Lorick KL Jensen JP Fang S Ong AM Hatakeyama S Weissman AM RING fingers mediate ubiquitin-conjugating enzyme (E2)-dependent ubiquitination Proc Natl Acad Sci U S A 1999 96 20 11364 11369 10.1073/pnas.96.20.11364 10500182 PMC18039

[B11] Huibregtse JM Scheffner M Beaudenon S Howley PM A family of proteins structurally and functionally related to the E6-AP ubiquitin-protein ligase Proc Natl Acad Sci U S A 1995 92 11 5249 7761480 10.1073/pnas.92.11.5249-b PMC55685

[B12] Aravind L Koonin EV The U box is a modified RING finger - a common domain in ubiquitination Curr Biol 2000 10 4 R132 R134 10.1016/S0960-9822(00)00398-5 10704423

[B13] Liu Z Oughtred R Wing SS Characterization of E3Histone, a novel testis ubiquitin protein ligase which ubiquitinates histones Mol Cell Biol 2005 25 7 2819 2831 10.1128/MCB.25.7.2819-2831.2005 15767685 PMC1061639

[B14] Liu Z Miao D Xia Q Hermo L Wing SS Regulated expression of the ubiquitin protein ligase, E3(Histone)/LASU1/Mule/ARF-BP1/HUWE1, during spermatogenesis Dev Dyn 2007 236 10 2889 2898 10.1002/dvdy.21302 17823942

[B15] Wang H Zhai L Xu J Joo HY Jackson S Erdjument-Bromage H Tempst P Xiong Y Zhang Y Histone H3 and H4 ubiquitylation by the CUL4-DDB-ROC1 ubiquitin ligase facilitates cellular response to DNA damage Mol Cell 2006 22 3 383 394 10.1016/j.molcel.2006.03.035 16678110

[B16] Kopanja D Roy N Stoyanova T Hess RA Bagchi S Raychaudhuri P Cul4A is essential for spermatogenesis and male fertility Dev Biol 2011 352 2 278 287 10.1016/j.ydbio.2011.01.028 21291880 PMC3065526

[B17] An JY Kim EA Jiang Y Zakrzewska A Kim DE Lee MJ Mook-Jung I Zhang Y Kwon YT UBR2 mediates transcriptional silencing during spermatogenesis via histone ubiquitination Proc Natl Acad Sci U S A 2010 107 5 1912 1917 10.1073/pnas.0910267107 20080676 PMC2836623

[B18] Iyengar PV Hirota T Hirose S Nakamura N Membrane-associated RING-CH 10 (MARCH10 protein) is a microtubule-associated E3 ubiquitin ligase of the spermatid flagella J Biol Chem 2011 286 45 39082 39090 10.1074/jbc.M111.256875 21937444 PMC3234733

[B19] Su AI Cooke MP Ching KA Hakak Y Walker JR Wiltshire T Orth AP Vega RG Sapinoso LM Moqrich A Large-scale analysis of the human and mouse transcriptomes Proc Natl Acad Sci U S A 2002 99 7 4465 4470 10.1073/pnas.012025199 11904358 PMC123671

[B20] Su AI Wiltshire T Batalov S Lapp H Ching KA Block D Zhang J Soden R Hayakawa M Kreiman G A gene atlas of the mouse and human protein-encoding transcriptomes Proc Natl Acad Sci U S A 2004 101 16 6062 6067 10.1073/pnas.0400782101 15075390 PMC395923

[B21] Ge X Yamamoto S Tsutsumi S Midorikawa Y Ihara S Wang SM Aburatani H Interpreting expression profiles of cancers by genome-wide survey of breadth of expression in normal tissues Genomics 2005 86 2 127 141 10.1016/j.ygeno.2005.04.008 15950434

[B22] Thorrez L Van Deun K Tranchevent LC Van Lommel L Engelen K Marchal K Moreau Y Van Mechelen I Schuit F Using ribosomal protein genes as reference: a tale of caution PLoS One 2008 3 3 e1854 10.1371/journal.pone.0001854 18365009 PMC2267211

[B23] Affymetrix Microarray suite users guide 2001 5 Affymetrix, Santa Clara

[B24] Shima JE McLean DJ McCarrey JR Griswold MD The murine testicular transcriptome: characterizing gene expression in the testis during the progression of spermatogenesis Biol Reprod 2004 71 1 319 330 10.1095/biolreprod.103.026880 15028632

[B25] Namekawa SH Park PJ Zhang LF Shima JE McCarrey JR Griswold MD Lee JT Postmeiotic sex chromatin in the male germline of mice Curr Biol 2006 16 7 660 667 10.1016/j.cub.2006.01.066 16581510

[B26] Tiwari S Weissman AM Endoplasmic reticulum (ER)-associated degradation of T cell receptor subunits. Involvement of ER-associated ubiquitin-conjugating enzymes (E2s) J Biol Chem 2001 276 19 16193 16200 10.1074/jbc.M007640200 11278356

[B27] Nian H Zhang W Shi H Zhao Q Xie Q Liao S Zhang Y Zhang Z Wang C Han C Mouse RING finger protein Rnf133 is a testis-specific endoplasmic reticulum-associated E3 ubiquitin ligase Cell Res 2008 18 7 800 802 10.1038/cr.2008.73 18574499

[B28] Neutzner A Neutzner M Benischke AS Ryu SW Frank S Youle RJ Karbowski M A systematic search for endoplasmic reticulum (ER) membrane-associated RING finger proteins identifies Nixin/ZNRF4 as a regulator of calnexin stability and ER homeostasis J Biol Chem 2011 286 10 8633 8643 10.1074/jbc.M110.197459 21205830 PMC3048745

[B29] de Bie P Ciechanover A Ubiquitination of E3 ligases: self-regulation of the ubiquitin system via proteolytic and non-proteolytic mechanisms Cell Death Differ 2011 18 9 1393 1402 10.1038/cdd.2011.16 21372847 PMC3178436

[B30] Petroski MD Deshaies RJ Function and regulation of cullin-RING ubiquitin ligases Nat Rev Mol Cell Biol 2005 6 1 9 20 15688063 10.1038/nrm1547

[B31] Romrell LJ Bellve AR Fawcett DW Separation of mouse spermatogenic cells by sedimentation velocity A morphological characterization. Dev Biol 1976 49 1 119 131 10.1016/0012-1606(76)90262-1 176074

[B32] Bellve AR Millette CF Bhatnagar YM O'Brien DA Dissociation of the mouse testis and characterization of isolated spermatogenic cells J Histochem Cytochem 1977 25 7 480 494 10.1177/25.7.893996 893996

[B33] Gan H Lin X Zhang Z Zhang W Liao S Wang L Han C piRNA profiling during specific stages of mouse spermatogenesis RNA 2011 17 7 1191 1203 10.1261/rna.2648411 21602304 PMC3138557

[B34] Nishito Y Hasegawa M Inohara N Nunez G MEX is a testis-specific E3 ubiquitin ligase that promotes death receptor-induced apoptosis Biochem J 2006 396 3 411 417 10.1042/BJ20051814 16522193 PMC1482824

[B35] Ogawa M Mizugishi K Ishiguro A Koyabu Y Imai Y Takahashi R Mikoshiba K Aruga J Rines/RNF180, a novel RING finger gene-encoded product, is a membrane-bound ubiquitin ligase Genes Cells 2008 13 4 397 409 10.1111/j.1365-2443.2008.01169.x 18363970

[B36] Locke M Tinsley CL Benson MA Blake DJ TRIM32 is an E3 ubiquitin ligase for dysbindin Hum Mol Genet 2009 18 13 2344 2358 10.1093/hmg/ddp167 19349376 PMC2694686

[B37] Bonifacino JS Dell'Angelica EC Springer TA Immunoprecipitation Curr Protoc Immunol 2001 8 8 3 10.1002/0471142735.im0803s41 18432858

[B38] Laney JD Hochstrasser M Analysis of protein ubiquitination Curr Protoc Protein Sci 2002 14 14 15 10.1002/0471140864.ps1405s29 18429222

