# Supplementary material for: Mining and characterization of ubiquitin E3 ligases expressed in the mouse testis
Source: BMC Genomics. 2012 Sep 19;13:495. doi: 10.1186/1471-2164-13-495 (PMC3460789; doi:10.1186/1471-2164-13-495)
Supplement: Additional file 1 — Summary of published papers about E3s in spermatogenesis. [file 1471-2164-13-495-S1.doc]

Summary of published papers about E3s in spermatogenesis

| **Abbreviation**  **of E3 ligase** | **Specie** | **Functional**  **domain** | **Expressed**  **primarily by** | **Function / mechanism** | **Journal** | **PMID** |
| --- | --- | --- | --- | --- | --- | --- |
| Cul4A- CRL4 complex | *c.elegan*  *mouse*  *et al* | RING | postnatal: gonocyte  pubertal: spermatogonia | meiotic progression | *TrendsBiochem Sci,* 2009 Nov; 34 (11) :562-70.  *Dev Biol,*2011 Apr 15;352(2):278-87 | 19818632  21291880 |
| LASU1 | *rat* | HECT | spermatogonia | histone ubiquitination | *Mol Cell Biol,*2005 Apr;25(7):2819-31.  *Dev Dyn,*2007 Oct; 236(10):2889-98. | 15767685  17823942 |
| Rnf8 | *human*  *mouse*  *et al* | RINGfinger | early spermatocyte stage | ubiquitinate H2A in DSB, remove nucleosome | *Cell,*2007 Nov 30;  131 (5):887-900.  *Dev Cell,*2010 Mar 16;18(3):371-84. | 18001825  20153262 |
| Rnf168 | *human*  *mouse*  *et al* | RINGfinger | early spermatocyte stage | recruit by Rnf8 and ubiquitinate H2A(X) | *BMC MolBiol,* 2009 Jun 5;10:55. | 19500350 |
| PHF9/FancL | *human*  *mouse*  *et al* | PHD finger | unknown | DNA damage  response | *Nat Genet,* 2003 Oct;35(2):165-70.  *Yi Chuan Xue Bao,* 2006 Jan;33(1):49-  55.  *Genes Cells,*2007 Mar;12(3):299-310. | 12973351  16450587  17352736 |
| BRCA1/BARD1  Complex | *human*  *mouse*  *et al* | RING | spermatogonia, pachytene  spermatocyte | apoptosis,  DNA repair | *Nat Genet*, 1996 Dec;14(4):430-40.  *Oncogene,* 1998 Jan 8;16(1):61-8.  *Biol Reprod,* 2004 Nov;71(5):1614-24 | 8944023  9467943  15240424 |
| Mei4 | *mouse*  *yeast* | RINGfinger | pachytotene spermatocyte , early spermatocyte | Meiotic Crossing Over and DSB formation. | *Genes Dev,*2010 Jun 15;24(12):  1266-80. | 20551173 |
| Hei10 | *human* | RING | -- | otholog with Mei4 | *PLoS Genet,* 2007 Aug;3(8):e139. | 17784788 |
| UBR1 | *rat*  *mouse*  *human*  *et al* | RINGfinger | Germ cell | seems no effect on fertility | *Pro Natl Acad Sci,*1998 Jul 7; 95 (14):7898-903.  [Nat Genet](http://www.ncbi.nlm.nih.gov/pubmed/16311597), 2005 Dec;37(12):1345-50. | 9653112  16311597 |
| UBR2 | *rat*  *mouse*  *et al* | RINGfinger | leptotene spermatocyte | transcription silence via ubiquitinate H2A,  necessary for chromosome stability. | [*Mol Cell Biol*,](http://www.ncbi.nlm.nih.gov/pubmed/14585983)2003 Nov;23(22):8255-71.  *Proc Natl Acad Sci*, 2010 Feb 2; 107 (5):1912-7.  [*PLoS One*,](http://www.ncbi.nlm.nih.gov/pubmed/22616001) 2012; 7 (5):e37414. | 14585983  20080676  22616001 |
| β-TrCP-SAG/ROC-SCFcomplex | *mouse* | RING  F-boxet al | spermatocyte | testes architecture | *Genes Dev,* 2010 Mar 1;24(5):470-7. | 20194439 |
| SAG/ROC-β-TrCP-SCF complex | *human*  *mouse*  *et al* | RING | -- | possible cell apoptosis | *Antioxid Redox Signal,*2001 Aug; 3(4):635-50.  *Neoplasia,* 2006 Dec;8(12):1042-54. | 11554450  17217622 |
| RNF32 | *human* | RING-H2 | spermatocyte, | unknown | *Biochem Biophys Res Commun,*2002 Mar 22;292 (1):58 -65. | 11890671 |
| ZNF645 | *human* | RINGfinger | spermatocyte  leydig cell | unknown | *Asian J Androl,* 2010 Sep;12(5):  658-66. | 20657603 |
| Siah1a | *mouse drosophila*  *et al* | RING | spermatocyte (possible) | required for male meiosis I | *Mol Cell Biol,*2002 Apr;22(7):2294-303. | 11884614 |
| rat100 | *rat* | HECT | pachytene spermatocyte | unknown | *Endocrinology,*  2002 Oct;143  (10):3740-7. | 12239083 |
| hyperplastic disc | *drosophila* | HECT | unknown | required for spermazoon formation | *Cell Biol Int*,2010 Oct 1;34(10):991-6. | 20604743 |
| Elfless | *Drosopila* | RING finger | tail cyst cell | cell apoptosis | *Fly*,2008 Nov-Dec; 2(6):269-79. | 19077536 |
| APC/C complex | *human*  *mouse*  *et al* | RING | metaphase of germ cell | chromosome segregation | *Annu Rev Cell Dev Biol*,2008;24:475-99. | 18598214 |
| Cullin3-Roc1b-KLHL10 complex | *human*  *mouse drosophila* | RING  BTB | elongating spermatid | spermatogenesis required, caspase activation | *Pro Natl Acad Sci*,  2004 May 18;101  (20):7793-8.  *BiolReprod*,2006 Jan;74(1):102-8.  *PLoS Biol*,2007 Oct;5(10):e251. | 15136734  16162871  17880263 |
| MARCH-11 | *rat* | RINGfinger | early spermatid | ubiquitinate SAMT in TGN  MVB pathway | *J Biol Chem*,2007 Aug 24;282(34):  24806-15. | 17604280 |
| Rnf133 | *mouse* | RINGfinger | elongating spermatid | involved in ERAD pathway | *Cell Res*, 2008 Jul;18(7):800-2. | 18574499 |
| MARCH10 | *rat* | RINGfinger | elongating spermatid | flagella formation  of spermatid | *J Biol Chem*,2011 Nov11;286(45):  39082-90. | 21937444 |
| Rnf19a | *rat* | RINGfinger | round spermatid | acrosome biogenesis (possible) | *Dev Dyn*,2009 Jul;  238(7):1851-61. | 19517565 |
| MEX | *mouse* | RINGfinger | unknown | promote Fas apoptosis | *Biochem J*,2006 Jun 15;396(3):  411-7. | 16522193 |
| HERC4 | *mouse* | HECT | spermatid | spermatozoa maturation | [*Genomics*,](http://www.ncbi.nlm.nih.gov/pubmed/15676274)2005 Feb;85(2):153-64.  *Dev Biol*,2007 Dec 15;312(2):501-8. | 15676274  17967448 |
| Rnf180/Rines | *mouse* | RINGfinger | unknown | unknown | *Genes Cells*,2008 Apr;13(4):397-409. | 18363970 |
| Itch | *Rat* | HECT | Sertoli cell | degrade occludin in tight junction | *J Cell Physiol*,2005 Jun;203(3):564-72. | 15605377 |
| Mkrn1 | *zebrafish,*  *chicken*  *et al* | RINGfinger | unknown | unknown | *BMC Genomics*,  2010 Dec 20;11: 721. | 21172006 |
