# Supplementary material for: Mining and characterization of ubiquitin E3 ligases expressed in the mouse testis
Source: BMC Genomics. 2012 Sep 19;13:495. doi: 10.1186/1471-2164-13-495 (PMC3460789; doi:10.1186/1471-2164-13-495)
Supplement: Additional file 5 — Expression of annotated E3s from iTRAQ database. [file 1471-2164-13-495-S5.doc]

Expression of annotated E3s from iTRAQ database

| Domain | Gene symbol | Chromosome location | Peptides ratio of ITRAQ | | | Expression level |
| --- | --- | --- | --- | --- | --- | --- |
|  |  |  | pacSC/  SG-A | rST / SG-A | eST / SG-A |  |
| **RING** | Bmi1 | 2 | 1.253 | 0.454 | 0.132 | P |
|  | Trim69 | 2 | 1.173 | 0.872 | 0.454 | SP |
|  | Zfp831 | 2 | 1.05 | 1.82 | 1.801 | A |
|  | Hltf | 3 | 1.302 | 1.699 | 1.179 | P |
|  | Rnf20 | 4 | 0.801 | 0.256 | 0.128 | P |
|  | Mtf2 | 5 | 0.869 | 0.575 | 0.631 | P |
|  | Baz1b | 5 | 0.867 | 0.609 | 0.445 | P |
|  | Brap | 5 | 0.99 | 0.722 | 0.61 | HP |
|  | Trim28 | 7 | 1.013 | 1.016 | 0.278 | MS |
|  | Pml | 9 | 0.497 | 0.163 | 0.133 | P |
|  | Arih1 | 9 | 0.736 | 1.429 | 1.672 | SP |
|  | Arih2 | 9 | 1.354 | 2.801 | 2.87 | HP |
|  | Vps11 | 9 | 0.873 | 0.64 | 0.705 | P |
|  | Fhl4 | 10 | 0.862 | 2.617 | 4.739 | SP |
|  | Mex3d | 10 | 0.762 | 4.901 | 8.191 | SP |
|  | Zbtb24 | 10 | 0.889 | 0.358 | 1.001 | P |
|  | Rnf146 | 10 | 1.683 | 2.445 | 2.287 | P |
|  | Pcgf2 | 11 | 0.166 | 0.121 | 0.078 | P |
|  | Mnat1 | 12 | 1.276 | 0.871 | 0.321 | P |
|  | Rnf17 | 14 | 1.315 | 0.733 | 0.403 | SP |
|  | Mll2 | 15 | 2.293 | 5.552 | 4.592 | P |
|  | Ring1 | 17 | 1.103 | 0.364 | 0.193 | P |
|  | Trim26 | 17 | 1.179 | 1.861 | 1.852 | P |
|  | Trim36 | 18 | 0.731 | 5.153 | 12.146 | SP |
|  | Zfpl1 | 19 | 0.895 | 1.035 | 0.741 | P |
| **HECT** | Trip12 | 1 | 0.912 | 0.7 | 0.596 | P |
|  | Herc6 | 6 | 57.221 | 102.061 | 85.434 | HP |
|  | Ube3a | 7 | 0.668 | 0.715 | 0.629 | P |
|  | Nedd4 | 9 | 0.611 | 0.168 | 0.106 | P |
|  | Herc4 | 10 | 0.98 | 1.085 | 1.602 | HP |
|  | Hectd1 | 12 | 0.705 | 0.521 | 0.341 | P |
|  | Ubr5 | 15 | 0.865 | 0.668 | 0.248 | HP |
|  | Huwe1 | X | 0.811 | 0.71 | 0.725 | P |
| **U box** | Sh3gl2 | 4 | 0.724 | 0.209 | 0.24 | P |
|  | Ube4b | 4 | 0.681 | 0.397 | 0.254 | P |
|  | Ift172 | 5 | 3.076 | 3.724 | 2.581 | SP |
|  | Stub1 | 17 | 1.58 | 1.722 | 1.204 | P |
|  | Prpf19 | 19 | 0.957 | 0.607 | 0.201 | P |
